# Primary cortical neurons form calcium-phosphate sheets with a bone-precursor-like ultrastructure

**DOI:** 10.1101/2025.08.04.668590

**Published:** 2025-08-06

**Authors:** Erik D. Anderson, Christopher A. Cronkite, Philip R. Baldwin, Carlota P. Abella, Joseph G. Duman, Ashleigh N. Simmonds, M. Neal Waxham, Kimberley F. Tolias, Steven J. Ludtke

**Affiliations:** 1 Department of Biochemistry and Molecular Pharmacology, Baylor College of Medicine, Houston, TX, USA; 2 Department of Neuroscience, Baylor College of Medicine, Houston, TX, USA; 3 Department of Neurobiology and Anatomy, McGovern Medical School, University of Texas Health Science Center at Houston, Houston, TX, USA

## Abstract

Calcium-phosphate is a critical component in healthy bone and teeth formation, but its pathologic buildup in brain can occur in dyshomeostatic calcium disorders like Alzheimer’s disease and Leigh syndrome. Extracellular calcium-phosphate in the nervous system is not well understood, but prior evidence suggests mitochondria as a potential source. Recently, large unidentified double-membrane-bound hyper-electron dense sheet aggregates of unknown content were reported in Huntington’s disease model neurons using cryo-ET that were absent in wild-type control neurons. We use a combination of cryo-ET, cryo-CLEM, and LDSAED to demonstrate that these sheet aggregates are generated by wild-type cortical neurons isolated from embryonic day 18 rat embryos and have an octacalcium phosphate-like structure. They appear to be derived from mitochondria and are extruded at least in part by migrasomes. These findings reveal an important link for how mitochondria can serve as reservoirs for intracellular and extracellular calcium-phosphate highlighting them as potential therapeutic targets in neurological disorders characterized by pathological calcification, such as Alzheimer’s disease.

## Introduction:

Calcium-phosphate (CaP) biomineralization is an important process that forms the inorganic component of bone and teeth. However, its deposition in the nervous system is also a prevalent symptom of brain pathology. It is seen in a wide variety of both rare and common diseases from inherited mitochondrial encephalopathies such as Leigh Syndrome to neurodegenerative disorders like Alzheimer’s disease (Tsolaki et al., 2022; Monfrini et al., 2023). CaP buildup in the brain can cause seizures (Loeb et al., 2006), but where exactly this CaP originates is an open and important question.

Although physiologic biomineralization processes such as bone formation offer valuable insights, the underlying sequence of events involved remains incompletely understood and is subject to ongoing debate. Increasing evidence supports bone formation beginning with an amorphous calcium-phosphate (ACP, Ca_9_(PO_4_)_6_ ) precursor that lacks long range structure, which either progresses through an octacalcium phosphate-like (OCP, Ca_8_(HPO_4_)_2_(PO_4_)_4_·5H_2_O) intermediate or directly converts into hydroxyapatite (HA, Ca_10_(PO_4_)_6_(OH)_2_) (Termine and Posner, 1970; Mahamid et al., 2011, 2008; Crane et al., 2006)). This process has been shown to occur in extracellular matrix vesicles released from osteoblasts (Hasegawa et al., 2017), but where the ACP originates is still debated. Molecules such as Annexin-related proteins (Thouverey et al., 2011) and poly(ADP-ribose) (Genge et al., 1990; Müller et al., 2019) that are enriched in matrix vesicles are thought to play an important role in calcium sequestration. However, the ACP granules that form in Ca_2_^2+^ and PO_4_^3−^ supersaturated mitochondria (Greenawalt et al., 1964) have also been hypothesized to contribute CaP through mitophagy-dependent transfer of these granules to autolysosomes (Boonrungsiman et al., 2012; Pei et al., 2018).

Anytime there are large, rapid calcium fluctuations in a cell, CaP precipitation is possible (Wuthier et al., 1985). In neutral to slightly alkaline environments like the mitochondrial matrix, when calcium and phosphate ions reach supersaturation, ACP is frequently the first precipitate to form (Duvvuri and Lood, 2021). High levels of magnesium, citrate, and other ions in the matrix inhibit hydroxyapatite conversion (Duvvuri and Lood, 2021; Brdiczka and Barnard, 1979), however it has been reported in the mitochondria of mouse neurons following excitotoxic shock (Maetzler et al., 2009). Hydroxyapatite has also been reported in the mitochondria of cardiomyocytes that have undergone ischemia-reperfusion injury, which occurs when blood flow is restored to previously oxygen-deprived tissue (Jennings, 2013).

Ischemia causes mitochondrial calcium, ADP, and pyrophosphate concentrations to increase and pH to drop through decreased oxidative phosphorylation and increased glycolysis (Halestrap et al., 2004). Following reperfusion, increased oxidative phosphorylation causes mitochondrial pH to rise. However, the concomitant high calcium concentration can induce the mitochondrial permeability transition pore (mPTP) to open, allowing calcium and other solutes <1.5 kDa to flow out of the matrix (Halestrap et al., 2004). Classically, severe metabolic disturbances trigger programmed cell death via apoptosis or necrosis. In contrast, less severe ionic and oxygen-related stress leads to mitophagy, the partial or complete engulfment of damaged mitochondria into autophagosomes (Rottenberg and Hoek, 2021). Interestingly an alternative mechanism for mitochondria quality control has recently been identified: some damaged mitochondria are actively extruded from cells in large (~3 μm) vesicles called migrasomes, through a process termed mitocytosis (Jiao et al., 2021). Migrasomes are released on the lagging edge of migrating cells and have been identified in primary mouse hippocampal neurons (Ma et al., 2015, 2023). They are distinct from exosomes, and some were shown to rupture and release their contents into the extracellular space (Ma et al., 2023), providing a mechanism for how damaged mitochondria and their fragments can be found outside of cells.

Similar reperfusion-like injuries are difficult to avoid when isolating embryonic neurons for cell culture. A standard procedure that our lab follows to isolate rat cortical neurons uses a base media of ~4° C Hanks’ balanced salt solution (HBSS) during benchtop dissection and ~37° C Neurobasal media during trituration and subsequent culture (Tolias et al., 2005; Duman et al., 2013). Low temperature and no calcium during dissection helps prevent ischemic damage following removal from maternal circulation by slowing oxidative phosphorylation, limiting NMDA receptor activation and keeping the mPTP closed (Pivovarova et al., 2004; Lemieux et al., 2017). However, upon transfer into warm Neurobasal media with a higher calcium concentration of 1.8 mM, this ion will rapidly enter the cell, providing a setting where ACP could precipitate.

Recently, large unidentified sheet aggregates were observed in Huntington’s disease model neuronal cultures that were absent in WT control neuron cultures prepared under the same conditions (Wu et al., 2023). These aggregates had a similar hyper-electron dense appearance to the ACP granules when visualized with cryo-electron tomography (cryo-ET), but with no apparent periodic structure. While the authors hypothesized that these aggregates were within autophagosomes or mitophagy-related vesicles and were possibly derived from damaged mitochondria, this was not confirmed. The aggregates’ molecular constituents remain unknown, and they have not been reported in normal healthy neurons.

We have observed what appear to be the same sheet aggregates in WT rat embryonic neuron cultures at both 1 and 20 days *in vitro* (DIV). In this study, we use a combination of cryo-ET, cryo-correlative light and electron microscopy (cryo-CLEM), and low-dose selected area electron diffraction (LDSAED) to characterize the aggregates. These methods have the advantage of resolving intracellular structures at nm resolution in a near-native state through millisecond vitrification in liquid ethane and visualization with transmitted electrons. Our results suggest these aggregates are CaP precipitated in mitochondria and are extruded at least in part by migrasomes. These results provide a mechanism for how mitochondria of cortical neurons can serve as a nexus for CaP precipitates in the central nervous system.

## Results:

**Figure 1.**
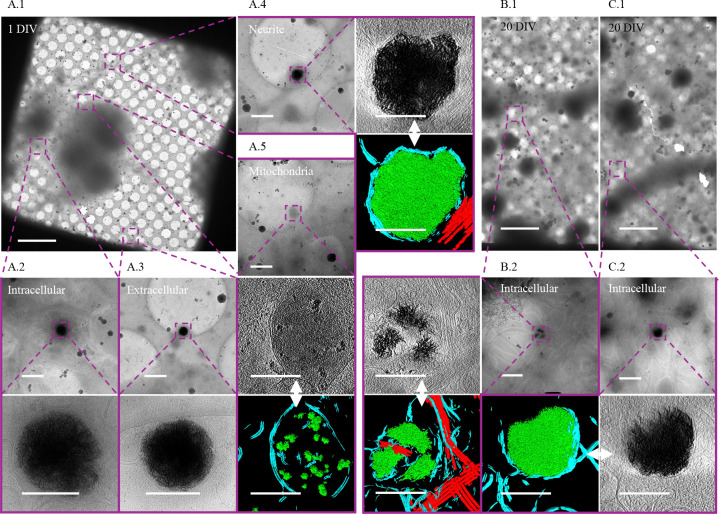
Sheet aggregates are present in both 1 DIV and 20 DIV rat cortical neurons. **(A.1)** 700× cryo-EM micrograph of a holey-carbon grid square depicting a typical vitrified cultured 1 DIV rat cortical neuron. **(A.2)** 5,300× (top) and high (bottom) mag cryo-EM micrograph images depicting aggregates within a soma. **(A.3)** 5,300× (top) and 45,000× (bottom) cryo-EM micrograph images showing an extracellular aggregate. **(A.4)** 5,300× cryo-EM micrograph (top left), 45,000× cryo-ET tomogram (top right), and 45,000× cryo-ET tomogram segmentation (bottom right) depicting aggregates within a neurite. **(A.5)** 5,300× cryo-EM micrograph (top), 45,000× cryo-ET tomogram (middle), and 45,000× tomogram segmentation (bottom) depicting mitochondria with calcium phosphate granules. **(B.1)** and **(C.1)** depict 700× cryo-EM micrographs of a typical vitrified cultured 20 DIV rat cortical neuron. **(B.2)** 5,300× cryo-EM micrograph (top right), 45,000× cryo-ET tomogram (top left), and 45,000× tomogram segmentation (bottom left) depicting aggregates. **(C.2)** 5,300× cryo-EM micrograph (top right), 45,000× cryo-ET tomogram (bottom right), and 45,000× tomogram segmentation (bottom left) depicting aggregates. Dashed boxes and lines depict areas of interest that are gradually increased in magnification. In segmentations, green = aggregates, cyan = membranes, and red = microtubules. Double-headed arrows depict corresponding tomogram and segmentation. Scale bars for 700× images = 10 μm; 5,300× = 1 μm; and 45,000× = 200 nm.

We isolated cortical neurons from E18 rat embryos and grew them on cryo-EM grids for 24 hours under conditions designed to optimize healthy growth. At this stage, viable neurons can be distinguished from non-viable neurons by the presence of extensive cytoplasm and the early formation of neurites (Fig. 1 A.1). In these cultures, we observe aggregates intracellularly (Fig. 1 A.2, A.4) in both cell bodies (Fig. 1 A.2) and neurites (Fig. 1 A.4) as well as extracellularly (Fig. 1 A.3). They appear to be in most neurons, though the number varies widely without any morphologic predictability (i.e., there are some neurons with abundant cytoplasm that have numerous aggregates, while others possess few or no aggregates). The same is true for neurons whose cytoplasm has retracted. Generally, there appear to be more aggregates in the soma than in neurites but calculating the abundance of aggregates/neuron or aggregates/cellular region is limited by the detection method. We chose cryo-transmission electron microscopic methods over room-temperature because cells are vitrified in a few milliseconds without necessary fixatives or stains that could affect the aggregate formation and structure. This may be why before Wu *et al.*’s 2023 study they were not reported*.* High resolution cryo-EM is currently the only method to identify them, but because it utilizes transmitted electrons, the thick center of a neuron’s soma makes it impossible to definitively know the total number in that area. While cryo-FIB/SEM can be used to thin these regions, the material above and below the section is lost in the process. Therefore, we cannot quantitatively assess abundance, but qualitatively they appear at some level in a clear majority of neurons imaged and are more prevalent in the soma and extracellularly than in neurites.

When viewed in 2D, as noted by Wu *et al*, the aggregates appear like fibers. However, with tomographic reconstruction and segmentation in 3D, it becomes clear that these apparent fibers are actually sheets (Fig. S1 A). The central region of more developed aggregates is generally amorphous in appearance and difficult to segment (Fig. 1 A.4). Aggregates such as these appear similar to the small calcium phosphate granules known to form in mitochondria (Fig. 1 A.3), though roughly 10x larger (sheet aggregates = ~ 200 nm – > 1 μm; granules = ~ 20 nm – 100 nm). After 24 hours all the aggregates appear enclosed in at least a double-membrane, consistent with Wu *et al.*’s observation in Huntington’s disease neurons (Wu et al., 2023), with some residing within multilamellar structures as well. In 20 DIV neurons, the aggregates remain readily detectable but take on a more variegated morphology (Fig. 1 B.1, B.2), and many appear smaller (Fig. S1 A). Additionally, some 20 DIV neurons appear to no longer be double membrane bound (Fig. S1 A). However, we do still observe aggregates similar to the 1-day aggregates (Fig. 1 C.1, C.2).

Wu *et al.* only detected aggregates in their Huntington’s disease model human induced pluripotent stem cells (iPSCs) and Huntington’s disease model primary mouse cortical neurons. They did not observe aggregates in WT control cells (Wu et al., 2023). To ensure the aggregates are not specific to our group’s isolation method, we viewed embryonic rat hippocampal neuron tilt-series produced and collected independently and were able to identify aggregates in 10 DIV neurons (Fig S1 B). While aggregates were less common in these tomograms, the images were collected for a publication (Fischer et al., 2018) focused on axons and dendrites, and so cell bodies and extracellular regions were intentionally avoided. Similarly, before our focus shifted to specifically studying aggregates, they appeared only occasionally, as we were primarily focusing on synapses, where they occur infrequently. Since most past Cryo-ET studies of neurons have been focused on the thinnest, easiest to image, regions, this may explain why aggregates have not been previously reported in WT neurons.

**Figure 2.**
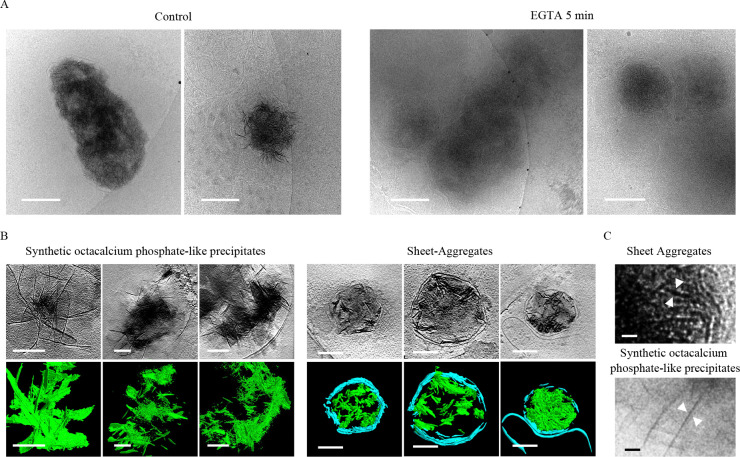
Sheet aggregates have a calcium-dependent structure and appear morphologically similar to OCP-like precipitates. **(A)** Cryo-ET tomograms and corresponding segmentations of 1 DIV cortical neurons incubated in low-magnesium artificial cerebrospinal fluid and either immediately frozen (left images) or incubated with 10mM of EGTA for 5 minutes (right images). For segmentations, green = aggregates; cyan = membrane. **(B)** Cryo-ET tomograms and corresponding segmentations depicting OCP-like precipitates and the aggregates. In segmentations, green = OCP-like precipitate/aggregates; cyan = membranes. **(C)** Magnified view of OCP-like and aggregate micrograph depicting their fibrous appearance in 2D. All scale bars = 200 nm except in (D), scale bars = 10 nm.

Establishing the exact chemical identity of the aggregates has required multiple strategies. Wu *et al.* noted they do not have any clear periodic structure in Fourier space or a similar structure to previously known protein aggregates like α-synuclein, β-amyloid, or Huntingtin. Additionally, they do not appear morphologically similar to any proteins known to form sheet structures such as clathrin and spectrin. The high electron density of the aggregates, similar to amorphous calcium-phosphate mitochondrial granules, indicates that a large fraction of the aggregate mass must be a high-Z element. We hypothesized that if Ca^2+^ is a major component of the aggregates, then chelation would cause some change in the aggregates. As a simple test, we chelated 1 DIV neuron media with 10 mM EGTA for 5 minutes prior to vitrification, and found that, despite the short timeframe, the sheet structure of many of the aggregates was ablated as compared to control neurons (Fig. 2 A). All micrographs were collected with the same electron dose and defocus. While the ablated aggregates still retained some amorphous hyper-electron dense material, the fibrous appearance in 2D is greatly reduced to the extent that identification even becomes difficult.

ACP is frequently the first CaP phase to precipitate in cellular environments like the mitochondria because, despite HA being favored in the slightly alkaline mitochondrial matrix pH of 7.8–8 (Wilson et al., 2021), ions such as magnesium and citrate inhibit its conversion (Duvvuri and Lood, 2021). However, if the Ca:P ratio is maintained at ~1.67, the pH >~7.0, and the inhibitory ion concentrations (e.g. Mg^2+^) permit, HA will form over time. If instead the ratio and pH drop to ~1.33 and ~5.5–7.4, OCP will form before HA, and if it continues to drop to 1:1 and <~5.5, a type of aggregate called brushite will precipitate (Habraken et al., 2013; Wuthier et al., 1985). OCP is striking because it forms ~1.85 nm thick plates that appear like sheets in 3D (Habraken et al., 2013). Wu *et al.* found their sheets to be ~2 nm thick, and we found a similar range for ours, though because the sheets often are not oriented perpendicular to the field of view and Z-resolution is diminished in tomograms, they can appear to be upwards of 7 nm. Based on this morphologic data, the EGTA experiment demonstrating calcium-dependence, and the aggregates’ striking hyper electron-dense resemblance to ACP mitochondrial granules, we hypothesized that these aggregates may represent OCP.

To test this hypothesis, we chemically synthesized OCP aggregates and performed cryo-ET for comparison with the cellular aggregates. Habraken *et al.* previously reported OCP-like structures form when CaCl_2_ is mixed slowly with K_2_HPO_4_ in Tris buffered saline at room temperature and pH 7.4 (Habraken et al., 2013). In the first ~20 minutes, dendritic-like pre-nucleation calcium triphosphate ([(Ca(HPO_4_)_3_]^4−^, Ca:P 0.30 – 0.40) complexes aggregate into polymeric strands/nodules (Ca:P 0.30 – 0.40). After ~20–60 minutes these rearrange into Ca^2+^-deficient amorphous calcium phosphate ([Ca_2_(HPO4)_3_]^2−^_,_ Ca:P ~0.67) spheres, then Ca^2+^-deficient OCP-like aggregated spheres and ribbons (15–110 minutes, Ca:P ~1.00), followed by elongated OCP-like plates (>3 hours, Ca:P ~1.33), and finally HA plates (1 month+, Ca:P ~1.67) (Habraken et al., 2013). The term “OCP-like”, rather than just “OCP”, is used because they contain all the characteristic diffraction peaks for OCP except the (100) peak. This is present when 1.85 nm thin crystal plates form; however, Habraken *et al* reported their plates were slightly thinner at ~1.4 nm. The calcium deficient ACP was reasoned to generate because it was ACP in solution, and it was noted that when dried, it had the characteristic Ca:P of ~1.5. This point emphasizes why cryogenic investigation of these aggregates is important, because how the sample is prepared can affect the analysis of calcium-dependent structures. We followed their procedure for 3 hours to enrich for OCP-like ribbons and plates. We then vitrified the samples, collected tomograms of identified precipitates, segmented the structures, and compared them to the aggregates.

Both the aggregates and OCP form fibrous strands in 2D, which are clearly sheets when segmented in 3D (Fig. 2 B). Though their fiber thicknesses appear similar, close inspection does reveal visual differences (Fig. 2 C). Some of the aggregates appear mottled, and generally the aggregate clumps are smaller. This is likely due to the fact that when formed in neurons, they are membrane-bound, which confines the size and direction the sheets can grow. Additionally, in the aggregates’ environment competing ions like Mg^2+^, carbonates, and other phosphates can bind, thereby affecting the sheet thickness by changing how planes stack.

**Figure 3.**
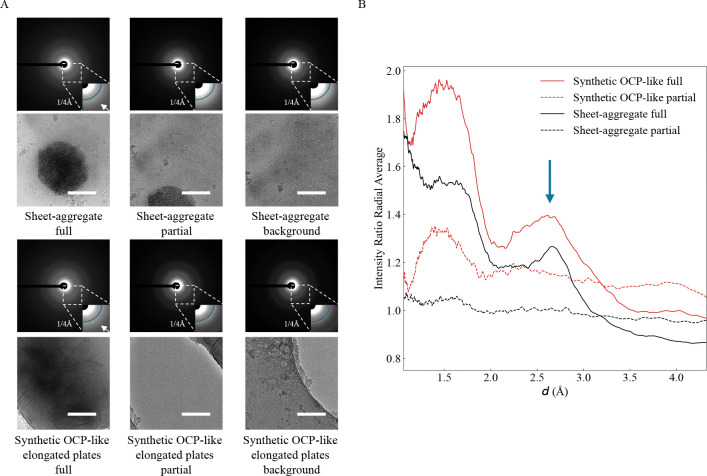
Sheet aggregates have a similar diffraction signature to OCP-like elongated plate HA precursors. **(A)** LDSAED of a sheet aggregate from 1 DIV rat cortical neurons and synthesized OCP-like elongated plates. Top image of pair is the electron diffraction micrograph depicting a maximum spatial frequency of 1/1.0 Å^−1^ in the full image and 1/2.4 Å^−1^ in the inset. Reference radial curve in the inset image corresponds to 1/4 Å^−1^. The white arrow is emphasizing the broad band between ~1/2.6 Å^−1^ – 1/3.3 Å^−1^ observed previously in OCP-like elongated plates (Habraken et al., 2013). Scale bars = 200 nm. **(B)** Ratio of radially averaged full or partial electron diffraction image intensities from (A) over each group’s respective background image. The x-axis has been converted to d spacing (1/q) and the arrow indicates the broad ~2.6–3.3 Å peak.

To confirm they have an OCP-like structure, we used LDSAED to compare the diffraction pattern of aggregates from 1 DIV neurons to the lab synthesized OCP-like elongated plates. Representative diffraction images for both samples show broad, diffuse rings consistent with poorly ordered/non-crystalline material (Fig. 3A). Habraken *et al.* previously reported OCP-like ribbons and elongated plates have a broad peak between ~2.6–3.3 Å that changes to a sharp peak at 2.8 Å as it converts to HA (Habraken et al., 2013). To investigate whether this is present in our data, we collected images with an aggregate centered in the image (“full”), where only a small amount of aggregate was visible (“partial”) and a nearby control background region. We then divided the radially averaged intensity distribution for the “full” and “partial” images by each sample’s corresponding background to dampen non-specific peaks such as those at ~3.71 Å and ~2.15 Å due to vitreous ice (Dowell and Rinfret, 1960). We found a broad peak ranging between ~2.5–3.2 Å present in the “full” image that overlaps well with our OCP-like elongated plate control (Fig. 3A, 3B) and to the previously reported 2.6–3.3 Å peak observed. This supports the sheet aggregates being HA precursor OCP-like plates. We next investigated where these aggregates specifically nucleate in neurons.

**Figure 4.**
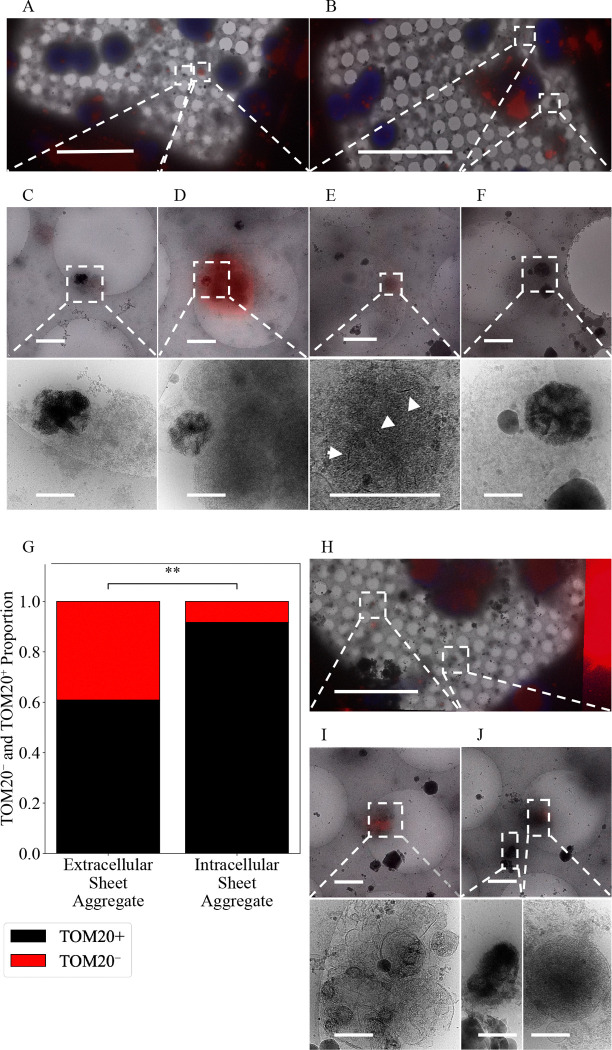
Sheet aggregates are well associated with TOM20^+^ mitochondria but poorly associated with COX4^+^ mitochondria. **(A)** and (**B)** 700× micrographs of PFA fixed vitrified 1 DIV rat cortical neurons overlaid with TOM20 (red) and Hoechst (blue) fluorescent stains. (**C)** 5,300× micrograph of extracellular aggregates with a weak TOM20 stain (top) and corresponding 45,000× image (bottom). **(D)** 5,300× micrograph of aggregates with a strong TOM20 stain near TOM20^+^ mitochondria (top) and corresponding 45,000× image (bottom). **(E)** 5,300× micrograph of aggregates forming within TOM20^+^ mitochondria (top) and corresponding 45,000× image (bottom), white arrowheads depict nascent sheets forming. **(F)** 5,300× micrograph of extracellular aggregates with no TOM20 stain (top) and corresponding 45,000× image (bottom). **(G)** Plot depicting the proportion of aggregates that are extracellular TOM20^+^ (n=28, 61%) or TOM20^−^ (n=18, 39%) versus the proportion that are intracellular TOM20^+^ (n=33, 92%) or TOM20^−^ (n=3, 8%). A two-sided Fisher’s exact test was used to determine significance; odds ratio = 0.141, p-value=0.001883, 95% CI = (0.05 – 0.56). ** = p-value ≤ 0.01. **(H)** 700× micrographs of PFA fixed vitrified 1 DIV rat cortical neurons overlaid with COX4 (red) and Hoechst (blue) fluorescent stain. **(I)** 5,300× micrograph of COX4^+^ extracellular mitochondria with calcium phosphate granules (top) and corresponding 45,000× image (bottom). **(J)** 5,300× micrograph of extracellular COX-negative aggregates and COX4^+^ mitochondria (top) and corresponding 45,000× image of aggregates (bottom left) and mitochondria (bottom right). Dashed boxes and lines depict areas of interest that are gradually increased in magnification. Scale bars for 700× micrograph = 25 μm; 5,300× = 1 μm; and 45,000× = 200 nm.

Given that many of the aggregates are found extracellularly 1 day following isolation, we hypothesized they are a deleterious CaP precipitate that the cells clear after ionic disequilibrium occurs. In line with this thinking, Wu *et al.* conjectured previously that the aggregates could be derived from damaged mitochondria undergoing mitophagy (Wu et al., 2023). To test if the aggregates are associated with mitochondria, we stained for a protein member of the translocase of the outer mitochondrial membrane complex (TOM) called TOM20. We achieved this by fixing cells in paraformaldehyde and performing immunofluorescent staining prior to freezing (Fig. 4 A–F). Upon visualization, we found many aggregates were associated with the TOM20 stain (Fig. 4 C–E) but not all (Fig. 4 F). Intriguingly, whether the aggregates were found intracellularly or extracellularly was a significant predictor of its TOM20 status. We found ~92% of intracellular aggregates identified stained positive for TOM20 versus only ~61% of extracellular aggregates (Fig. 4G). Additionally, we found nascent aggregates forming within visually identified mitochondria (Fig. 4 E), providing strong evidence for mitochondrial origin, though we cannot exclude the possibility of other additional sources. Next, we immunostained neuron cultures for the electron transport chain protein COX4 (Fig. 4 H–J) and found that mitochondria both with and without calcium-phosphate granules stain positive (Fig. 4 I and J). However, the aggregates do not appear associated with COX4 (Fig. 4 J). The findings that most intracellular aggregates stain positive for TOM20 and nascent sheets can be found in mitochondria support the hypothesis that at least some aggregates are mitochondrial in origin. However, the result that they do not stain positive for respiratory proteins like COX4 suggests mitochondria with aggregates are no longer functional.

As previously discussed, Wu *et al.* hypothesized the aggregates may be derived from mitochondria undergoing mitophagy (Wu et al., 2023). To test for mitophagy involvement, we stained for lysosomes using Lysotracker^™^ as well as the ATG8 family protein LC3, which is a ubiquitous marker for autophagy/mitophagy during protein cargo recruitment and autophagosome-lysosome fusion (Uoselis et al., 2023). We did not detect the aggregates spatially associated with lysosomes or LC3 (Fig. S2 A–F). Because the aggregates do not appear to be within lysosomes or LC3^+^ autophagosomes, given many are found extracellularly and migrasomes have been shown to remove damaged mitochondria (Jiao et al., 2021), we hypothesize they may be extruded through mitocytosis.

Recently, the metalloprotease CPQ was identified as a highly specific marker for migrasomes that differentiates them from other extracellular vesicles like exosomes (Zhao et al., 2019). We stained for CPQ in our 1 DIV cortical neurons and identified aggregates inside CPQ^+^ extracellular vesicles (Fig. 4 E, F, and H). We also found CPQ^+^ vesicles containing mitochondria (Fig. 4 G), which supports the previous finding that migrasomes undergo mitocytosis (Jiao et al., 2021). CPQ was not associated with all extracellular aggregates (Fig. 3 I and J). However, in these cases the aggregates no longer appeared to be inside in any larger migrasome-appearing vesicle, implying they may be byproducts of migrasomes that have released their contents (Ma et al., 2023).

Knowing the aggregates are likely CaP precipitates that form in mitochondria and given that shocks of high calcium can cause precipitation events, we investigated whether incubation in media with a lower calcium concentration would decrease their size. We incubated cells in Neurobasal (NB) or F-12 media +/− Fetal Bovine Serum (FBS) after standard isolation since F-12 has a lower calcium (0.3 mM vs 1.8 mM), glucose (10 mM vs 25 mM), and glutamine (1 mM vs 2 mM) concentrations. We also tested the effect that time in culture has on aggregate formation. To investigate their size, we collected >90 2D micrographs of aggregates from all conditions and measured their area. We found that the aggregates in neurons from the 1 DIV F-12 + 10% FBS media and 20 DIV NB media conditions appear to be significantly smaller than the other groups (Fig. 4 A). Interestingly, the decreased area in the 1 DIV F-12 + 10% FBS and 20 DIV NB conditions were different in character from each other. Aggregates from the F-12 condition neuron cultures appeared within membrane boundaries of similar size to the control conditions; however, the aggregates themselves were less dense, leading to a smaller area value (Fig. 1 B). Conversely, aggregates from 20 DIV control neurons appeared similarly dense but overall smaller in size than those found in the 1 DIV control neurons (Fig. 1 B). These data indicate that neuronal media affects the size of the aggregates and longer time in culture increases the number of small aggregates.

## Discussion:

We have observed the presence of hyper-electron-dense sheet aggregates in both 1 and 20 DIV embryonic rat cortical neurons. They appear both intra and extracellularly, as well as within both neurites and cell bodies. Calcium chelation with EGTA ablates the sheet morphology, but retains high contrast, strongly implicating Ca^2+^ as a critical component of the complex. Electron diffraction analysis further supports the identity of the aggregates as OCP-like plates via a broad band in the ~2.5–3.2 Å range matching lab-grown CaP aggregates. Together these experiments provide strong evidence that these aggregates are primarily composed of CaP in an OCP-like state.

To assess the cellular origin of the aggregates, we first observed that in both 1 DIV and 20 DIV primary cortical neurons they appear intracellularly, extracellularly, in neurites, and cell bodies, and retain a mitochondria-like appearance and size. After 1 day all the aggregates appeared to be at least double membrane bound, however some aggregates in the 20 DIV group do not show a discernable encapsulating membrane. We stained 1 DIV neurons for the outer membrane mitochondrial protein TOM20 and found many of the aggregates associated with this protein. However, these aggregates do not appear significantly associated with the electron transport chain protein COX4, indicating they may be non-energetically active/damaged mitochondria or mitochondria-derived vesicles. They also do not appear associated with the ATG8 family LC3 protein involved in autophagosome recruitment or lysosomes. However, they are associated with CPQ^+^ extracellular vesicles called migrasomes, which may serve as one of the cell’s mechanisms for extracellular export. Culturing neurons for 1 day in F-12 media + 10% BSA, which contains a decreased Ca^2+^ concentration, or for 20 days in NB media causes the aggregates to decrease in size relative to those in 1 DIV neurons cultured in NB. These findings support the hypothesis that following the metabolically stressful isolation procedure, primary cultured rat cortical neurons form mitochondria-derived OCP-like precipitates.

Formation of these aggregates appears to vary significantly with culturing conditions and buffer. While we can only speculate, this does offer a plausible explanation for why Wu *et al.* detected similar aggregates in human and mouse Huntington’s disease cortical neurons but not their corresponding WT control neurons (Wu et al., 2023). Human iPSC neural progenitors, derived from fibroblasts, do not undergo the same challenging isolation procedure. It is less clear why they were not observed in their mouse neuronal cultures, but some differences in our culture conditions do exist. Their embryonic mouse neurons were incubated in Neurobasal + 10% FBS and GlutaMAX^™^ for a day before switching to serum free media for 13 more days before cryopreservation. Our normal culture protocol uses 2mM glutamine without any serum incubation. While we found 10% FBS after 1 day with Neurobasal does not significantly decrease the aggregate size, we did not follow those cultures for 13 days. Given our findings that longer times in culture and post-isolation media with lower Ca^2+^ concentration cause the aggregates to decrease in size, it is plausible that anything that changes the calcium, phosphate, pH, or inhibitory ion concentration of the media during or following isolation will impact aggregate solubility and structure. Overall, our findings suggest that while Huntington’s disease likely causes an increased presence of these aggregates, they are not limited to this disease state as previously proposed.

It is well established that ACP granules can precipitate within mitochondria (Jasielec et al., 2020; Greenawalt et al., 1964). However, the presence of OCP-like structures in mitochondria remains more controversial, as some have argued that mitochondrial ionic conditions are not conducive to OCP formation (Jasielec et al., 2020). Despite this, the striking morphological and structural similarities between the intracellular aggregates we observed and the laboratory synthesized OCP-like material presents a compelling case. While definitive elemental and structural characterization, such as cryo-energy dispersive X-ray spectroscopy (cryo-EDX) or electron energy loss spectroscopy (cryo-EELS), could further strengthen this interpretation, access to these techniques for cryo-preserved specimens is currently limited to a small number of international sites and room temperature protocols are highly perturbative.

Our findings fit well into previous studies that investigated *in vivo* CaP formation in the nervous system. Hydroxyapatite was reported in the mitochondria of mouse neurons from fixed cortical tissue following excitotoxic shock (Maetzler et al., 2009), and microcalcification in Alzheimer’s disease patient neurons was associated with phosphorylated Tau presence (Tsolaki et al., 2022b). These demonstrate hydroxyapatite can form *in vivo* under pathologic conditions, but these and the vast majority of past studies were done using structure-altering fixatives and stains to view tissue samples at room temperature. In recent years however, cryo-FIB/SEM opens the possibility of investigating brain tissue and slice cultures in a near-native environment, and we predict as this method is increasingly used these OCP-like aggregates will be more widely observed.

Mitochondria have been implicated as a source of CaP during bone formation (Boonrungsiman et al., 2012; Pei et al., 2018). Extending this concept to neurons, our findings reveal that large OCP-like aggregates, substantially larger than the typical mitochondrial CaP granules, can form within mitochondria and are capable of being exported into the extracellular space. These results suggest a potential mechanism linking mitochondrial dysfunction to brain calcification disorders. Although our observations are in neurons, they bear striking parallels to CaP dynamics in osteoblasts during bone mineralization. This raises the intriguing possibility that brain calcification may arise, at least in part, from aberrant activation of pathways normally involved in bone growth. More broadly, our findings suggest that metabolically stressed neurons can act as a nucleation site for CaP accumulation, providing a mechanistic explanation for its extracellular deposition in neuropathologies such as the β-amyloid plaques of Alzheimer’s disease (Schober et al., 2021). These insights further support growing interest in targeting mitochondrial dysfunction as a therapeutic strategy in neurological diseases.

## Methods

### Neuron Culture and Media Experiments

E18 Long-Evans rats were used for cortical neuron cultures and experiments were approved by the Institutional Animal Care and Use Committees at Baylor College of Medicine. Neuron extraction was performed as previously described (Tolias et al., 2005; Duman et al., 2013). 1 day prior to isolation 2–4 grids were placed in 35 mm Matsunami glass bottom culture dishes (Avantor Sciences) dishes and UV-sterilized in the cell culture hood for 30 minutes. Then, 0.1 mg/mL poly-D lysine (Thermo Fisher) was added to the dish and incubated overnight at 37° C 5% CO_2_. Then next day, the poly-D lysine was removed the plates were washed 3× with phosphate buffered saline (PBS). After isolation, neurons were cultured at 37° C and 5% CO_2_ in Neurobasal media (Thermo Fisher) supplemented with 50X B27 (Thermo Fisher), 2mM L-glutamine (Thermo Fisher) and 10,000 U/mL Penicillin-Streptomycin (Thermo Fisher).

To test the effect of fetal bovine serum (FBS) and F-12 media, following isolation cells were either incubated with 10% FBS (Thermo Fisher), or in F-12 media (Thermo Fisher) with 10% FBS. Cells incubated for 24 hours did not have their media exchanged prior to freezing. Cells incubated for 20 days underwent a full media exchange 24 hours after plating, and then every 3–4 days 1/3^rd^ of the media was removed and replaced with fresh media. To test EGTA chelation, 23 hours after plating cells were incubated in low magnesium 0.2 μm-filtered 1.5X artificial cerebrospinal fluid (aCSF) pH 7.4 for 1 hour, containing 186 mM NaCl, 4.5 mM KCl, 1.0 MgCl_2_, 15 mM D-glucose, 15 mM HEPES, 4.5 mM CaCl_2_ (all reagents from Sigma-Aldrich). 10mM EGTA was then added for 5 minutes and neutralized by removing the media, washing 3X with aCSF and replacing with fresh media. Neurons were then frozen within 10 minutes of stimulation.

### Sample Vitrification

Neurons were frozen in liquid ethane cooled by liquid nitrogen using the Leica EM GP plunge freezer. Owing to transfer, room temperature incubation prior to freezing is unavoidable but was limited to less than 10 minutes. The chamber was set to 60% humidity, and grids were back blotted for 8 seconds if in 24-hour group and 15 seconds if in the 20-day group. 1.5 μL of 1:1 fresh Neurobasal media:cultured Neurobasal media was added to the front of the grid prior to blotting, except for EGTA stimulation where just fresh aCSF was used. Octacalcium phosphate precipitates were vitrified on R 1.2/1.3 Cu 200 mesh Quantifoil grids. The grids were glow discharged first using the PELCO easiGlow^™^ with 15 mAmps for 30 seconds, a 15 second hold time, and 0.4 mBar pressure. The grids were similarly frozen with the Leica EM GP plunge freezer except 3 μL of solution was added to the front of the grid and back blotted for 8 seconds. All grids were clipped with autogrid rings and C-clips (Thermo Scientific and Ted Pella) and stored in liquid nitrogen until imaging as described below.

### Cryo-Electron Microscopy and Tomography Collection

Frozen clipped neuron grids were imaged using a 200kV Glacios^™^ (Thermo Scientific), Falcon 4i direct electron detector (Thermo Scientific), and Tomography 5 software (Thermo Scientific). For cryo-ET, tilt series were acquired with an exposure of ~1.2–1.8 e^−^/Å^2^/tilt and total dose of ~61–91 e^−^/Å^2^/tilt series. We used a dose symmetric collection scheme ranging between −50° and +50° with 2° tilt step between −3 μm to −7 μm defocus and at 3.12 Å/pixel or 1.24 Å/pixel. EMAN2 was used to reconstruct tomograms using the pipeline described previously (Chen et al., 2019). Segmentations were created using EMAN2’s semi-automatic convolutional neural network method described previously (Chen et al., 2019). Segmentations were manually cleaned up in ChimeraX (Pettersen et al., 2021) using their Hide Dust and Map Eraser plugins to remove noise and false positives generated by neural network. Each segmentation and corresponding tomogram for display were then Gaussian filtered with a width of 20 using ChimeraX’s Map Filter plugin. For the EGTA chelation experiment, images were also collected on the Glacios^™^ with 5 e^−^/ Å^2^, −5 μm defocus, and 3.21 Å/pixel for each image. The experiment was repeated twice and grids from two different cultures for each condition (EGTA chelation and aCSF control) were imaged.

### Immunofluorescent Staining

Neurons were grown as described above for 24 hours, however prior to vitrification cells were fixed and stained. When staining for lysosomes, prior to fixation we incubated with LysoTracker^™^ (Thermo Fisher, L12492) at 50 nM for 30 minutes at 37° C and 5% CO_2_. To fix cells, we removed the Neurobasal media and washed 1× with PBS with calcium and magnesium (Thermo Fisher). We then fixed the cells in 4% formaldehyde (Sigma-Aldrich) diluted in PBS for 15 minutes at room temperature. Cells were then washed 3× with PBS and permeabilized for 10 minutes at room temperature using PBS containing 0.25% Triton X-100 (Sigma-Aldrich) and washed again 3× with PBS. Next, cells were blocked for 1 hour at room temperature with 5% bovine serum albumin (BSA, Sigma-Aldrich) in PBS and stained with the primary antibody overnight diluted in the 5% BSA blocking solution at 4°C (antibody information below). The next day, cells were washed 3× with 0.1% Tween-20 (Sigma-Aldrich) in PBS with 5-minute incubation between each wash, and then the secondary antibody was added, diluted in the blocking solution, and incubated at room temperature for 1 hour away from light. The cells were then washed 3× with PBS + 0.1% Tween-20, again with 5-minute incubations between washes, then washed 1× with regular PBS, and then vitrified as described above.

We used the following primary and secondary antibodies and dilutions. Primary antibodies: Rabbit TOMM20 [EPR15581–54] 1:250 (Abcam, ab186735); Rabbit COX IV 1:200 (Abcam, ab153709); Mouse LC3B [G-9] 1:100 (Santa Cruz Biotechnology, sc-376404); Rabbit PBCP/CPQ 1:100 (Proteintech, 16601–1-AP). Secondary antibody: Goat anti-rabbit IgG Alexa Fluor^™^ 565 nm 1:500 (Thermo Fisher, A11011); Donkey anti-mouse IgG Alexa Fluor^™^ 647 nm 1:500 (Thermo Fisher, A32787). To stain nucleic acids, we used Hoechst 33342 at 1 μg/mL (Thermo Fisher, 62249).

### Immunofluorescent Imaging:

Fixed and frozen neurons were imaged using the Aquilos 2 Cryo-Focused Ion Beam/Scanning Electron Microscope (cryo-FIB/SEM) equipped with the Integrative Fluorescent Light Module (iFLM) (Thermo Scientific). For this experiment only the SEM and iFLM were used. Grids were loaded into the microscope using the 35° shuttle and mapped with the MAPS 3.24 software (Thermo Scientific) using 195× magnification, 2kV voltage and 13pA current. When an ideal region was identified on the SEM map, the grid was imaged with the iFLM using the iFLM 1.3 software. The 385 nm channel was used to collect the Hoechst stain with an intensity of 10% and exposure time of 50 ms. The 565 nm channel was used for the Alexa Fluor^™^ 565 nm secondary antibody with 20% intensity and 50 ms exposure time. A reflection image for alignment was also collected using 1% intensity and 0.1 ms exposure time. A z stack of images was taken using a slice distance of 0.7 μm and the stack was summed into a maximum intensity image used for identification. 1–3 regions were collected per grid depending on how many sufficient regions were identified. Regions were then overlaid with the SEM map using the program’s semi-automatic alignment method. Then the grid was transferred to the Glacios^™^ for high-resolution imaging. An overview image at a magnification of 28.3 μm/pixel was taken and overlaid onto the SEM/fluorescent image in the MAPs program using the Hoechst nuclear stain as anchor points. Fluorescent puncta of interest were then identified and imaged in the Glacios^™^ at a pixel size of 3.2 Å/pixel with 10 e^−^ total dose and −10 μm defocus, and a search magnification of 13.5 Å/pixel and −85 μm defocus. When quantifying the proportion of intracellular vs extracellular aggregates that are TOM20^+^, we used a two-sided Fisher’s exact test to determine significance.

### Aggregate Area Calculation

To calculate aggregate areas, neurons were grown in each condition and vitrified as described under the “Neuron culture and media experiments” and “Sample vitrification” methods sections. Using the Glacios^™^ we collected ~30 micrographs of aggregates from 3 different cultures of each condition at a defocus of −10 μm, 3.21 Å/pixel, and 10 e^−^/image. Segmentation maps were manually generated using EMAN2 over each aggregate area (Dang and Ludtke, 2022). Using custom python scripts each mask was multiplied by the original image so only the aggregate pixel area remained. Each aggregate’s area within the image was then binary thresholded based on the equation: (*maximum pixel value* – *minimum pixel value*) – *pixel standard deviation ×* 1.5 for that specific area. E.g., if 2 aggregates were in the same image, a different threshold value for each aggregate was determined based off its pixel distribution. This was chosen empirically by visualizing all the images with different threshold levels and determining what maintained the most aggregate pixels over background. The area was determined using the Python skimage.measure function regionprops, converted to nm^2^, and graphed. Statistical significance was calculated using the Wilcoxon rank-sum test and p-values were corrected for multiple comparison using Benjamini-Hochberg correction.

### Octacalcium Phosphate Precipitation

To precipitate octacalcium phosphate (OCP) we followed the procedure previously reported by Habraken *et al*. (Habraken et al., 2013). Briefly, a 30 mL working buffer of 50mM Trizma^®^ base (Sigma) and 150 mM NaCl (Sigma) was made with 18.2 MΩ·cm ultrapure water and adjusted to pH 7.4 with 0.1 M NaOH (Sigma) and 0.1M HCl (Sigma). Next, 25 mL 14 mM K_2_HPO_4_ and 5 mL 25 mM CaCl_2_•H_2_O solutions were created in the Tris salt buffer and pH adjusted to 7.4 with 0.1 M NaOH. Then, the CaCl_2_ solution was added to the 25mL KH_2_PO_4_ solution at a rate of 10 μL/min for 3 hours at 21° C under constant 200 stirring. The pH was titrated with 0.1 M NaOH and 0.1 M HCl during the process to maintain a pH of 7.4. After 3 hours, the solution was added to grids, vitrified, and imaged as described in the “Sample vitrification” and “Cryo-electron microscopy and tomography collection” methods sections.

### Low Dose Selected Area Electron Diffraction

Cryo-selected area electron diffraction (SAED) was performed using a 200_k_V Glacios^™^ (Thermo Scientific) equipped with a Ceta-D camera (Thermo Scientific). We used a camera distance of 670 mm (Nyquist spatial frequency = 1.75 Å^−1^), a SA aperture size of 10 μm, and a defocus of −0.5 μm. Non-diffraction images of areas were also collected using a pixel size of 1.62 Å/pixel and a defocus of −2 μm. 1 DIV neuron aggregates and 3-hour OCP-like elongated plates were imaged in an incremental manner beginning directly over the object and gradually moving off to capture how the pattern changes as the target aggregate or OCP is no longer centered. Diffraction patterns were analyzed using EMAN2 (Tang et al., 2007) and custom python scripts. Specifically, diffraction images were centered, radially averaged, and 1/q weighted. To correct the background an adjacent image to the target (e.g. aggregate or OCP-like elongated plate) was collected, and the target radial distribution was divided by this distribution.

## Supplementary Material

Supplement 1

## Figures and Tables

**Figure 5.**
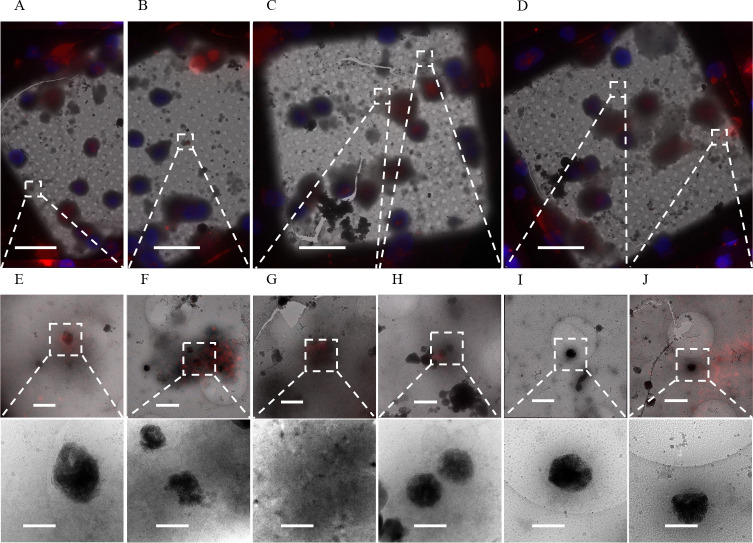
Sheet aggregates are found in CPQ^+^ migrasomes. **(A-D)** 700× cryo-EM micrographs of PFA fixed vitrified 1 DIV rat cortical neurons overlaid with CPQ (red) and Hoechst (blue) fluorescent stains. **(E)**, **(F)**, and **(H)** 5,300× cryo-EM micrographs depicting aggregates inside CPQ^+^ migrasomes (top) and their corresponding 45,000× images (bottom). **(G)** 5,300× cryo-EM micrograph depicting mitochondria inside CPQ^+^ migrasomes (top) and its corresponding 45,000× images (bottom). **(I)** and **(J)** 5,300× cryo-EM micrographs depicting extracellular aggregates not associated with CPQ^+^ migrasomes (top) and their corresponding 45,000× images (bottom). Dashed boxes and lines depict areas of interest that are gradually increased in magnification. Scale bars for 700× micrograph = 20 μm; 5,300× = 1 μm; and 45,000× = 200 nm.

## Data Availability

Representative tomograms presented in this article were uploaded to the Electron Microscopy Data Bank (EMDB). 1 DIV aggregate tomograms: EMD-71853, EMD-71859, EMD-71860, EMD-71861; 1 DIV mitochondria with ACP granules: EMD-71852; 20 DIV aggregate tomograms: EMD-71850, EMD-71851, EMD-71862, EMD-71863, EMD-71864; OCP precipitates: EMD-71865, EMD-71867, EMD-71868. All files for this project, e.g. the raw data for the cryo-ET, cryo-CLEM, and LDSAED experiments as well as Python scripts used for analysis, can be found on the Electron Microscopy Public Image Archive (EMPIAR) at EMPIAR-12905.

## Abbreviations

ACP: amorphous calcium phosphate
ATG8: autophagy-related protein 8 (family)
BSA: bovine serum albumin
CaP: calcium phosphate
COX4: cytochrome c oxidase subunit 4
CPQ: carboxypeptidase Q
cryo-CLEM: cryo-correlative light and electron microscopy
cryo-EM: cryo-electron microscopy
cryo-ET: cryo-electron tomography
DIV: *days in vitro*
E18: embryonic day 18
EGTA: ethylene glycol-bis(β-aminoethyl ether)-N,N,N′,N′-tetraacetic acid
F-12: Ham’s F-12 nutrient mixture
F-actin: filamentous actin
FBS: fetal bovine serum
FIB/SEM: focused ion beam / scanning electron microscopy
HA: hydroxyapatite
HBSS: Hanks’ balanced salt solution
iPSC: induced pluripotent stem cell
LC3: microtubule-associated protein 1 light chain 3 (ATG8 family member)
LDSAED: low-dose selected-area electron diffraction
mPTP: mitochondrial permeability transition pore
NB: Neurobasal (medium)
OCP: octacalcium phosphate
PFA: paraformaldehyde
TOM20: translocase of the outer mitochondrial membrane 20-kDa subunit
