# Supplementary material for: Primary cortical neurons form calcium-phosphate sheets with a bone-precursor-like ultrastructure
Source: bioRxiv. 2025 Aug 6:2025.08.04.668590. Preprint. [Version 1] doi: 10.1101/2025.08.04.668590 (PMC12340797; doi:10.1101/2025.08.04.668590)
Supplement: Supplementary file 1 [file NIHPP2025.08.04.668590v1-supplement-1.pdf]

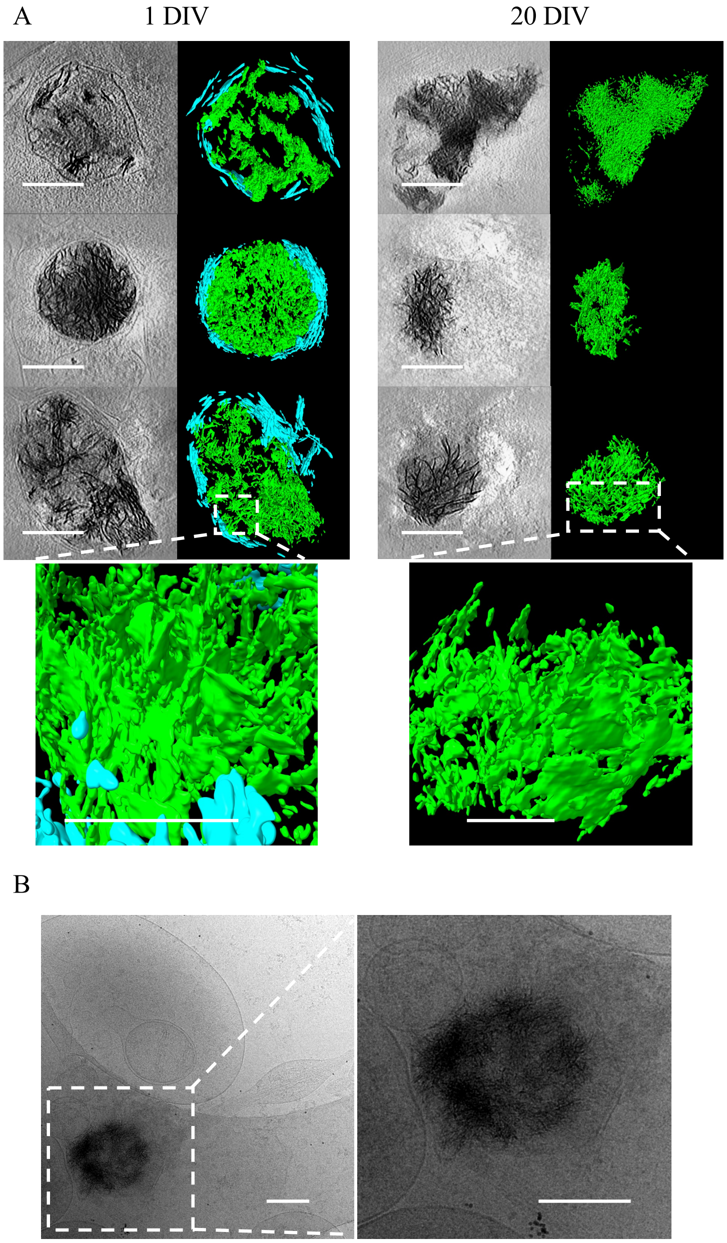

Figure S1. Sheet aggregates show filamentous morphology in 2D and sheet morphology in 3D. (A) Cryo-ET tomograms and corresponding segmentations of different kinds of aggregates in 1 DIV rat cortical neurons (left column images) and 20 DIV rat cortical neurons (right column images). Bottom images in each column depict a magnified side view of the sheet morphology in regions delineated by the dashed boxes. In the segmentations, green = aggregates; cyan = membranes. **(B)** Zero tilt micrograph of an aggregate found in a 10 DIV cultured hippocampal neuron. The tilt-series was collected independent of our group for a previously published study (Fischer et al., 2018), demonstrating the aggregates are not unique to our isolation/culturing method. All scale bars = 200 nm except for the bottom magnified side view images in (A), where scale bars = 100 nm.

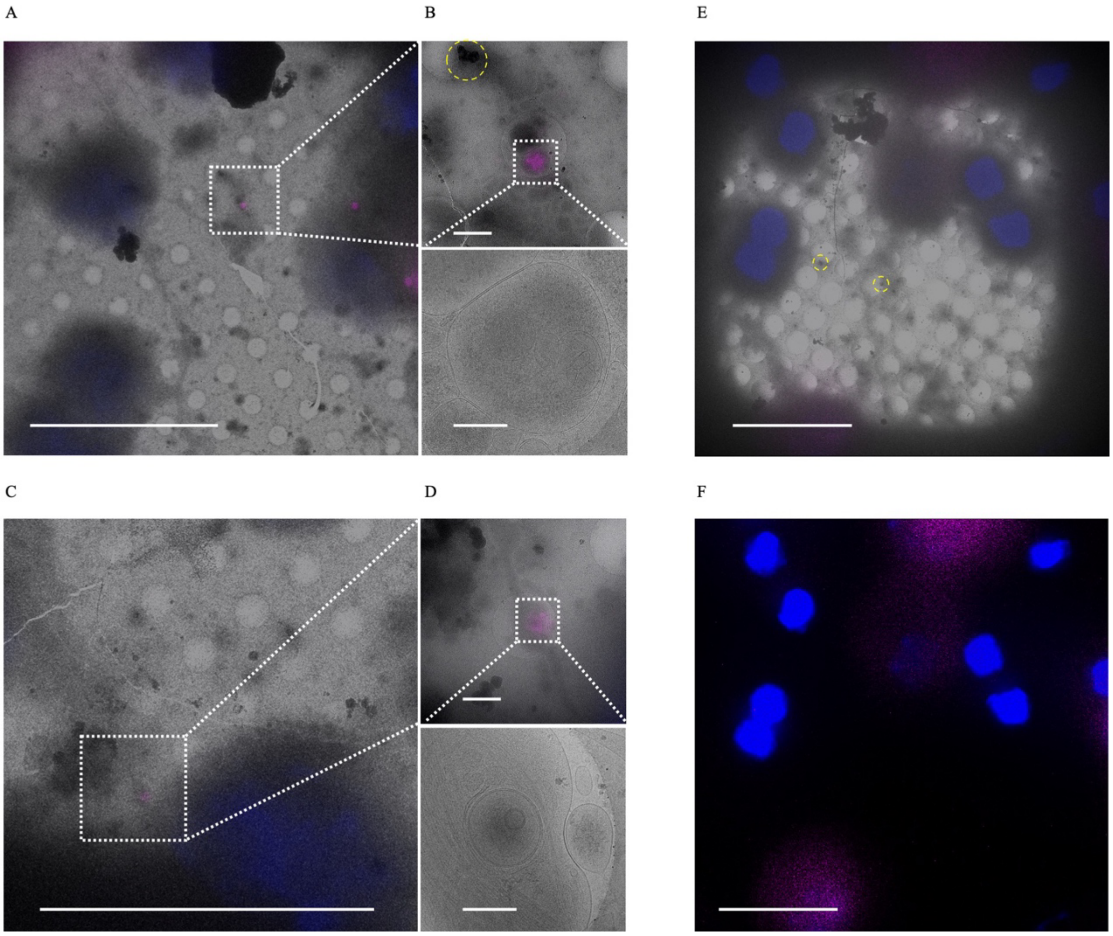

Figure S2. Sheet aggregates are not associated with lysosomes or LC3^{+} autophagosomes. (A) and **(C)** 700× cryo-EM micrographs of PFA fixed rat cortical neurons overlaid with Lysotracker™ (purple) and Hoechst (blue) fluorescent stains. **(B)** and **(D)** 5,300× cryo-EM micrographs of lysosomes overlaid with Lysotracker™ and Hoechst stains (top images) and their corresponding 45,000× micrographs (bottom

images). **(E)** 700× cryo-EM micrographs of PFA fixed rat cortical neurons overlaid with LC3 (purple) and Hoechst (blue) fluorescent stains. Dashed boxes and lines depict areas of interest that are gradually increased in magnification. **(F)** Same region as **(E)** except depicting the fluorescent signal alone without the micrograph overlay to emphasize the lack of LC3 puncta in a 700× micrograph with aggregates. Yellow dashed circles are outlining aggregates. Scale bars for 700× micrograph = 20 µm; 5,300× = 1 µm; and 45,000× micrograph = 200 nm.

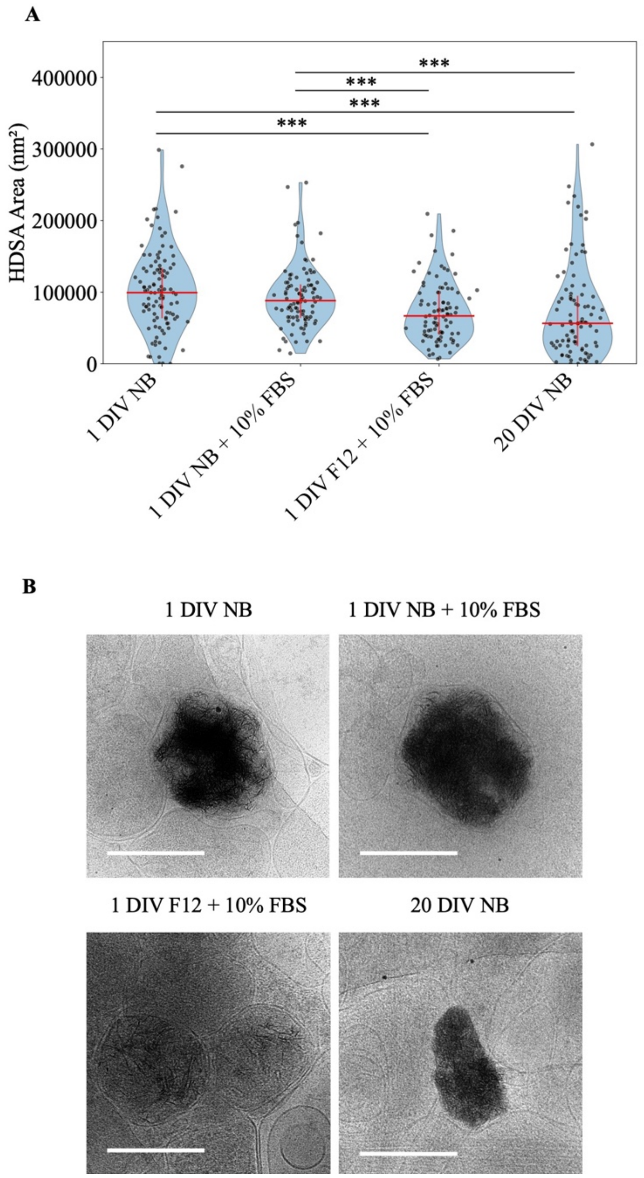

Figure S3. Sheet aggregate area decreases with F-12 media and time in culture. (A) Graph depicting area of aggregates in nm2 across different conditions. For each condition grids from 3 different cultures were imaged. 1 DIV Neurobasal (NB) media, n = 107; 1 DIV; Neurobasal (NB) + 10% FBS n = 100; 1 DIV F-12 + 10% FBS n = 94; 20 DIV NB n = 94; Median and interquartile range depicted in red. Wilcoxon rank-sum test followed by Benjamini-Hochberg correction for multiple comparison was done. *** = $p <0.001$, ** = $p < 0.01$, * = $p < 0.05$. **(B)** Representatives of median sized aggregates: 1 DIV NB median = 99,318 nm^2; 1 DIV NB+ 10% FBS, median = 88,134 nm^2; 1 DIV F-12 + 10% FBS, median = 66,782 nm^2; 20 DIV NB, median = 56,475 nm^2. All scale bars = 200 nm.
